# Is material deprivation decreasing in Germany? A trend analysis using PASS data from 2006 to 2013

**DOI:** 10.1186/s12651-018-0244-x

**Published:** 2018-10-05

**Authors:** Hans-Jürgen Andreß

**Affiliations:** Universität zu Köln, Cologne, Germany

**Keywords:** Deprivation, Trend analysis, Panel data, German Panel Study Labor Market and Social Security, PASS, Basic income support, Absolute poverty, D12, D31, I32

## Abstract

The analysis uses seven waves from the German Panel Study Labor Market and Social Security (PASS) covering the period from 2006 to 2013. During the observation period, Germany experienced a significant increase in average real incomes and employment, accompanied by a decrease of absolute income poverty as measured by the at-risk-of-poverty rate anchored at a fixed moment in time. PASS collects information on material deprivation with a list of 26 possessions and activities. The article discusses the difficulties of measuring material deprivation and identifies several sources of measurement error and selection bias. However, even when controlling for such errors and biases, regression models for different deprivation indices show a significant downward trend in material deprivation for the German resident population. Given the simultaneous decrease in absolute income poverty, it is concluded that measures of material deprivation behave like absolute income poverty indicators if the list of possessions and activities is not updated to changed living standard standards in society. A similar downward trend is observed also for individuals receiving basic income support. Moreover, supported individuals report deprivation differently depending on survey mode and number of previous panel interviews, raising the question of measurement equivalence in this subgroup.

**Electronic supplementary material:**

The online version of this article (10.1186/s12651-018-0244-x) contains supplementary material, which is available to authorized users.

## Introduction

Measures of material deprivation are an essential part of Eurostat’s regular statistical reporting on income and living conditions. The indicator is defined “as the percentage of population with an enforced lack of at least three out of nine material deprivation items in the ‘economic strain and durables’ dimension” (Eurostat 2018). Trend analyses of material deprivation often find a long-term decline of corresponding deprivation measures, even when controlling for socio-economic changes of the population (Berthoud and Bryan 2011; Figari 2012; Groh-Samberg and Goebel 2007). In Germany, according to Eurostat data, this indicator dropped from 13.5% in 2006 to 9.7% in 2016 (Eurostat 2018). Such a drop in material deprivation in the 2000s and 2010s is surprising given increasing poverty trends in the same period using either income or consumption as poverty indicators (Grabka and Goebel 2017; Hörstermann 2016). However, what is different from earlier periods is a significant increase of employment with mandatory social insurance coupled with a similarly large decrease of registered unemployment (Bundesamt et al. 2016, pp. 126–149). Earlier studies have shown that rising unemployment results in a positive deprivation trend (Andreß 2006).

Income and consumption poverty as relative poverty measures have been criticized for their inability to cope with upswings in living conditions. Since their poverty thresholds are connected to the center of the distribution of incomes or consumption expenditures (either the arithmetic mean or the median), they will always report a certain amount of poverty if income or consumption is sufficiently unequally distributed, even if incomes or expenditures are rising on average. In fact, if one fixes the income poverty threshold to the value observed in 2000, one observes—controlling for changes in consumer prices—a decreasing trend of income poverty from 15% in 2005 to 13.8% in 2012 (Goebel et al. 2015). This “at-risk-of-poverty rate anchored at a fixed moment in time” (Eurostat; for short the fixed AROP) is more in line with the observed trend in material deprivation.

Similarly, the list of items (the basket of commodities) that is used to evaluate material deprivation is not updated over time and hence, is also a poverty measure that is fixed in time. Some scholars have argued (e.g., Groh-Samberg and Goebel 2007) that the decrease in material deprivation may be due to certain items whose prices or availability may have changed due to either technological or socio-economic changes in society (such as significant improvements in housing conditions in Eastern Germany after German reunification). Moreover, collecting information about material deprivation is as difficult as collecting information on income and consumption and therefore, the decreasing trend may also be a result of measurement error, e.g., if respondents increasingly give positive answers the more often they are asked for their possessions and activities.

Up to now, measures of material deprivation have been mostly used in cross-sectional studies (Gordon and Pantazis 1997; Mack and Lansley 1985; Nolan and Whelan 1996; Pantazis et al. 2006). It has been shown that measures of low income and of material deprivation do not identify the same people as being poor, concluding that income is possibly a weak indicator of poverty and that people in persistent poverty are better identified by deprivation measures (Whelan et al. 2001, 2003, 2004). However, little is known about the long-term measurement characteristics of the deprivation approach. This article contributes to the latter question by asking whether this methodology can be used to validly describe long-term trends of deprivation.

But before we can answer this question we have to make sure that decreasing (or increasing) trends in deprivation are not due to different socio-economic compositions of the populations being compared over time. Hence, the test should not be based on global trends of deprivation (like the Eurostat figures in the beginning), but on trends that control for the main socio-economic correlates of deprivation, such as individual income and employment status (e.g. see Andreß 2006). Moreover, measurement equivalence is a prerequisite to compare deprivation scores over time.

This article uses seven waves (2006–2013) from the German Panel Study Labor Market and Social Security (PASS) to analyze the trend of deprivation during a period of 7 years. PASS is the best data source to analyze deprivation trends in Germany, because it collects on a yearly basis comprehensive information on 26 different possessions and activities, many more than the nine items that are used in official statistics of Eurostat and governmental poverty reports (for Germany see Bundesministerium für Arbeit und Soziales, o.J.). Nevertheless, the use of repeated observations of the same individuals over time (instead of pooling observations of different individuals from different cross-sections over time) poses additional problems if not all individuals continue to participate in the panel and drop-out is socially selective.

We first introduce the PASS data and its collection of material deprivation in Sect. 2, because this information is necessary to understand possible measurement and sample selection errors, which we discuss in Sect. 3. Based on the testing strategy proposed in Sects. 3, 4 describes our methods. Section 5 describes the test results. We will show that deprivation decreases in Germany in the observation period, even if one controls the socio-economic profiles of the respondents as well as possible measurement and selection errors during the process of data collection. Section 6 concludes with a discussion of possible explanations for this surprising result.

## Measures of material deprivation in the PASS data

PASS is a central dataset for research on labor market, poverty and means-tested income support in Germany. Established by the Institute for Employment Research (IAB) of the German Federal Employment Agency (BA) in 2006, annual surveys are conducted in households in receipt of basic income support [Unemployment Benefit II (UBII); Sample 1] and in households registered as residents of Germany (Sample 2). Initially, interviews are carried out with the heads of all selected households (termed reference person in the following). Subsequently, all members of each household aged 15 or over are interviewed (FDZ IAB, o.J.). In order to cope with panel attrition and population changes new UBII recipients are sampled in each new wave. Moreover, replenishment samples were taken in 2011 for UBII recipients (Sample 7)^1^ and households registered as residents of Germany (Sample 6).

Data were collected using a mix of computer-assisted telephone interviews (CATI) and computer-assisted personal interviews (CAPI). According to the user guide “[t]he mixed-mode design was chosen as a cost effective way of addressing various issues related to low income and welfare populations […]. Particular problems faced when trying to interview these groups are, for example, their tendency to relocate more frequently than the general population, difficulties in contacting them by phone due to low landline coverage, or changes in mobile phone numbers” (Trappmann et al. 2013, p. 16).

All samples collect information on material deprivation by asking the reference persons first whether the household owns certain possessions^2^ (13 items) and then whether the reference person or the whole household does certain activities^3^ (13 items). The complete list of all 26 items can be found in Table S1 of Additional file 1. They cover different life domains: leisure and social activities (4 items), solvency (5), health care (2), personal belongings (3), household equipment and devices (6), and housing and living environment (6). If the reference person gave a negative answer, he or she was asked whether the missing possession or activity was due to financial or due to other reasons.

Based on the assumption that household incomes determine economic well-being equally for all household members, analyses of income poverty assign equivalized household incomes to all members of the household and then analyze data at the person level using data of all interviewed household members. Data on material deprivation are also collected at the household level and we proceed in a similar way and assign possessions and activities as reported by the reference person to all household members. The following data analysis is done at the level of persons (and not households). The clustering of data within households may lead to an underestimation of standard errors, but is ignored in our analysis similar to many other analyses of household panel data.^4^

**Fig. 1:**
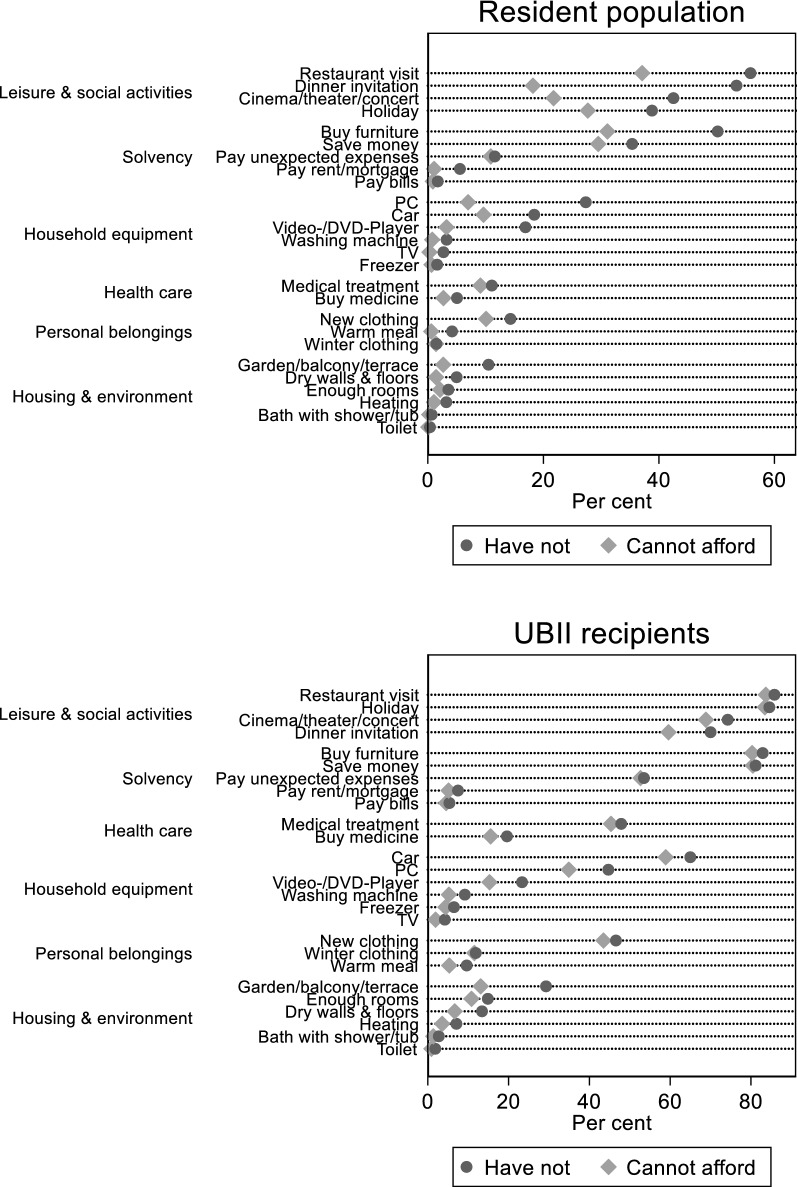
Percentage of respondents not having the item (“Have not”) and not being able to afford the item (“Cannot afford”). Data: Panel Study Labor Market and Social Security (PASS) samples 1, 2, 6, and 7; waves 1–7, 2006–2013. Weighted analyses controlling for survey design, selection probabilities, and non-response

As Fig. 1 shows, lacking items (termed “have-nots”) occur less often in the resident population then in the population of UBII recipients. If one counts only items lacking for financial reasons (termed “unaffordable items”), percentages get smaller but the differences between UBII recipients and the general population become even larger. Not surprisingly, the association between have-nots and unaffordable items is higher for UBII recipients than for the resident population. Leisure and social activities are most often missing (on average 26% in the resident population and 74% among UBII recipients), followed by solvency (21%, 46%), health care (8%, 34%), and household equipment items (12%, 25%). Personal belongings (7%, 23%) as well as housing and environmental items (4%, 12%), on the other side, are available in most cases, at least in the resident population.

## How to explain trends of deprivation over time?

The enforced lack of certain possessions and activities that are deemed necessary in society, termed deprivation by Townsend (1979), is usually interpreted as a result of insufficient resources and behaviors of the individual (and its household). Income is one, but not the only resource that contributes to the household’s living situation (Andreß 1999). As Ringen (1988) puts it, income is an indirect measure of poverty, while deprivation is a direct one. We distinguish between (i) individual behaviors as well as (ii) individual, (iii) household and (iv) external resources. (i) Individual behaviors that are relevant in this context (e.g., to be economical) are the topic of nutritional science and home economics. Unfortunately, information on these behaviors is hardly available in population surveys (and also in the PASS waves of our analysis).^5^ (ii) Examples of individual resources include earnings, education and employment status as well as gender and age. (iii) Household resources include (equivalized) household incomes and all the other resources of the individuals one is keeping house with (often operationalized by a household typology). Many of these resource indicators (ii) and (iii) simultaneously control different individual and household related needs. Finally, regionally different public infrastructures, labor markets and economic conditions provide (iv) external resources. If any of these behaviors and resources changes significantly over time, changes of deprivation can be expected. Hence, it does not make sense to compare overall deprivation trends. Instead, we want to compare these trends for individuals having identical behaviors and resources. Given we have sufficiently controlled for all these differences, we usually would not have any reason to believe that deprivation is increasing or decreasing over time.

H1: After controlling for individual behaviors as well as individual, household and external resources average material deprivation is constant over time.

On the other hand, measuring material deprivation is not an easy task and as the former section showed, the PASS instrument is quite voluminous (26 items, 3 dropped after wave 4), including a lot of qualifications (to whom each item pertains) and follow-up questions (asking for the cause of lacking possessions and activities). Therefore, it takes a lot of time both for interviewers and respondents to collect all necessary information, especially if respondents lack certain possessions and activities. Moreover, poor people may feel stigmatized or ashamed if they have to report year after year how deprived they are, especially so if their living situation deteriorates.

Research has shown that follow-up items immediately after the relevant filter produce answers to the filter question that avoid the follow-up question (Eckman et al. 2014; Kreuter et al. 2011). This would imply for the PASS instrument that once respondents know the instrument they are inclined to not report a missing item in the next panel wave, because then they would have to report why this is the case. This would increase the duration of the interview. Depending on survey mode and interviewer remuneration, also interviewers may have an interest in shortening interview duration by avoiding positive answers to filter questions that initiate a long series of follow-up questions (Josten and Trappmann 2016).

Whether respondents economize on interview time can be checked by comparing individuals that are acquainted with the instrument with those who are not. Therefore, we expect that.

H2a: Members of the two PASS refreshment samples (samples 6 and 7) report higher deprivation in 2011 than members of the two original samples (samples 1 and 2)

who in the same year have already been interviewed max. four times. Based on H2a, economizing on interview time is assumed to result in increasing underreporting of deprivation, which provides an alternative explanation compared to the one given in H1. However,

H2b: the assumed difference between the refreshment and the original samples in 2011 should gradually diminish in the following years as the members of the refreshment samples get to know the instrument.

Finally, if economizing implies that respondents learn to evade the time-consuming follow-up questions, then this effect should equally apply to an analysis of have-nots and unaffordable items. Respondents will simply report that they own (entertain) the particular possession (activity).

Economizing on interview time may also be pandered by survey mode. For respondents, CAPI and CATI imply more or less the same incentives when answering the deprivation instrument. However, for interviewers, CATI mode provides fewer opportunities to fake the interview, while in CAPI mode they may do the interview in such a way that complicated survey instruments do not take too much time. Assuming that from the beginning interviewers are informed about the instrument and how to economize on it, one would expect a time-constant difference between both survey modes.

H3: Interviews conducted in CAPI mode are subject to more underreporting of deprivation than interviews conducted in CATI mode.

However, if respondents being interviewed in CAPI mode are more deprived than those interviewed in CATI mode (mixed-mode interviewing was chosen for low income and welfare populations due to their high residential mobility, fewer landline telephones, and often changing mobile phone numbers; see Sect. 2), H3 is offset by positive selection bias:

H4: Respondents interviewed in CAPI mode are more deprived than respondents interviewed in CATI mode.

We expect this selectivity to be larger in samples from the resident population, where deprived individuals are clear outliers, while in samples from the population of UBII recipients they are not so different from the average sample member. As far as this selection is related to low income, it can be controlled by household income. However, if additional selection mechanisms come into play (e.g., high mobility, difficult telephone access, deprivation), CAPI will have an independent effect on deprivation.^6^

The estimated effect of survey mode in the sample ($\hat{\beta }_{2}$ in the following regression model) will measure the net effect of H3 *and* H4. Depending on the size of both effects H3 *and* H4, $\hat{\beta }_{2}$. may be positive, negative, or zero. This makes a test of the single hypotheses difficult. If selection into CAPI mode is due to time-constant characteristics of the respondents, e.g., when CAPI is used only for the long-term poor/deprived, one could control this potential problem by estimating models that only use the over-time within-person variation of the variables (by using fixed effects models). This, however, is not very plausible, since survey mode changes quite frequently during the observation period.^7^

However, deprived respondents may react differently to the instrument over time. They may embellish their actual situation because they feel ashamed or stigmatized about their deprived living standard. It is also possible that deprived individuals put up with their sub-standard living situation and accept it as normal. In all these cases they are more likely to report items missing for other than financial reasons or in the extreme case contend that they own them. In other words: They adapt their preferences to their factual living situation (Halleröd 2006).

H5: If adaptive preferences play a role we expect a downward trend of deprivation with the duration of UBII recipience.

This downward trend should be especially visible when focusing on the unaffordable items, but not so much when analyzing the have-nots which also include items missing for other reasons. In our later analysis we will estimate separate models for the general population and for UBII recipients and we expect H5 to be an important response behavior among UBII recipients.

A final alternative explanation to a decreasing time trend could be panel attrition: Individuals with low incomes and/or high material deprivation may leave the panel earlier than others, leaving behind—year after year—an increasingly positively selected data set. In the Socio-Economic Panel Study (SOEP) it has been found that drop-out is much higher for individuals with low incomes, but it can be controlled by re-weighting the remaining individuals in the panel (Rendtel et al. 1995). Therefore, one would expect that

H6: a decreasing time trend of material deprivation is less pronounced for weighted than for unweighted data, if the weights control for panel attrition.

One could also focus on individuals with complete over-time information only, i.e., a balanced panel of individuals participating in all waves. If one observes declining material deprivation in this subgroup, it cannot be explained by panel attrition. However, this alternative test is potentially biased because continuous participation in the panel may also be selective.

In sum, H2, H5, and H6 make predictions about different response behaviors and sample structures over time that provide alternative explanations for a change of deprivation (with and without controls) over time. H3 and H4 hypothesize that deprivation is related to survey mode. Hence, if the use of, say, CAPI interviews is increasing over time this would also result in a change of deprivation over time.

## Operationalization and statistical methods

Our analyses use the two original samples 1 and 2 and pool them with the two replenishment samples (samples 6 and 7), i.e., respondents that compared to individuals from samples 1 and 2 have not seen the deprivation instrument before. For reasons of simplicity, we ignore the UBII refreshment samples.^8^ The comparison of the original and the replenishment sample within both subgroups (German residents, UBII recipients) allows a test of H2. Moreover, in case of UBII recipients, we only include respondents in our analysis that receive UBII payments at the time of the interview.^9^ Without this selection, the group of UBII recipients would also include respondents that received UBII payments only in former waves but not the present one, i.e., respondents that have escaped from welfare dependency. It would be no surprise that their deprivation decreases.

### Dependent variable: deprivation

As explained in Sect. 2, the reference person reports for each item whether the household owns a particular possession or does a particular activity. In the negative case he or she has to report why the particular item is missing. For our analysis we assign possessions and activities as reported by the reference person to all household members. Since deprivation is defined as “enforced lack” (e.g., a missing car because it is too expensive and not because someone prefers a bike or public transport), most researchers compare individuals with items lacking for financial reasons in relation to individuals having the corresponding item (ignoring individuals lacking the item for other reasons). Let *F*_*ij*_ denote the dichotomous variable indicating whether individual *i* has item $j \left( {F_{ij} = 0} \right)$ or whether he or she lacks the item for financial reasons (*F*_*ij*_ = 1). Some researchers, however, argue that “(a) lack of an item is itself associated with low income, regardless of the ‘cannot afford’ criterion, and (b) respondents’ statement that they cannot afford an item is socially as well as economically constructed” (Berthoud and Bryan 2011, p. 154; McKay 2004). Therefore, other researchers ignore the follow-up question on financial or other causes and analyze the dichotomous variable *L*_*ij*_ indicating whether individual *i* does not have item $j \left( {L_{ij} = 1} \right)$ or owns item (*L*_*ij*_ = 0).^10^ The underlying measurement model assumes that the probability of a positive answer on either *F*_*ij*_ or *L*_*ij*_ is a result of individual *i*’s deprivation *θ*_*i*_, a latent variable being observed only indirectly via the answers to all items. In case of *F*_*ij*_, this latent structure model would be defined as follows (the model for *L*_*ij*_ would look alike):

1 $$\Pr \left( {F_{ij} \, = \,1 |a_{j} ,b_{j} ,\theta_{i} } \right)\, = \,\frac{{{ \exp }\left\{ {a_{j} \left( {\theta_{i} \, - \,b_{j} } \right)} \right\}}}{{1 + { \exp }\left\{ {a_{j} \left( {\theta_{i} \, - \,b_{j} } \right)} \right\}}}$$

One can think of (1) as a factor model for dichotomous variables (the famous Rasch model (Andrich 1988) is a special case). Estimation of the model needs a distributional assumption about the latent variable *θ*, which can be assumed to be either continuous or categorical (Heinen 1996).

The measurement model assumes that the $j = 1, \ldots , 26$ items are differently indicative of the underlying latent variable *θ*_*i*_: (a) They may be more or less “costly” in the sense that the more *i*'s deprivation *θ*_*i*_ exceeds the “costs” *b*_*j*_ of item *j* the higher the probability that item *j* is lacking. (b) They may discriminate more or less quickly between a positive and a negative answer as measured by the parameter *a*_*j*_. If item *j*. has large discriminatory power, *a*_*j*_ is larger and even small differences between individual deprivation *θ*_*i*_ and costs *b*_*j*_. of item *j* lead to a positive answer. It can be shown that a sum index of all missing items is a sufficient statistic for model (1), if the discrimination parameter *a*_*j*_ is the same for all items (Andreß 2008; Cappellari and Jenkins 2004; or any textbook on item response theory). In other words, in this case the ad-hoc method used in many deprivation analyses, simply counting the number of lacking items, is legitimate. For reasons of simplicity, we follow this research practice although—taking the sample of the resident population (Sample 2) as the reference—the hypothesis of equal discriminatory power of all items has to be rejected.^11^

In the following we ignore the three items dropped after wave 4. We compute four different (partly weighted) sum indices as follows:

2 $$S_{i}^{*} \, = \,\mathop \sum \limits_{j = 1}^{23} g_{j} \cdot s_{ij} .$$

*g*_*j*_ is a suitable weight and *s*_*ij*_ represents the dichotomous variables *F*_*ij*_ and *L*_*ij*_. If *F*_*ij*_ or *L*_*ij*_ is missing, the corresponding item is ignored in the summation. More specifically, the four indices measure.

- *S*_*i*_^*F*^, the number of missing items for financial reasons (*g*_*j *_= 1, *s*_*ij*_ = *F*_*ij*_),
- *S*_*i*_^*N*^, the number of missing items for financial reasons weighted by their perceived necessity *n*_*j*_ in the population (*g*_*j*_ = *n*_*j*_, *s*_*ij*_ = *F*_*ij*_),
- *S*_*i*_^*P*^, the number of missing items for financial reasons weighted by their prevalence *p*_*j*_ in the population (*g*_*j*_ = *p*_*j*_, *s*_*ij*_ = *F*_*ij*_), and
- *S*_*i*_^*L*^, the number of missing items for whatever reason (*g*_*j*_ = 1, *s*_*ij*_ = *L*_*ij*_).

*S*_*i*_^*F*^ is the usual deprivation index used in official statistics on material deprivation. *S*_*i*_^*N*^. is based on the assumption that the lack of necessary items is more depriving than the lack of unnecessary items (Halleröd 1995), while *S*_*i*_^*P*^ assumes that the lack of items that most people have is more depriving than the lack of less prevalent items (Desai and Shah 1988). Finally, *S*_*i*_^*L*^ accounts for the possibility of biased answers to the cause question by simply counting the have-nots (McKay 2004). Perceived necessities *n*_*j*_ and prevalences *p*_*j*_ in the population were derived for each item from the sample of the resident population (Sample 2).^12^

If adaptive preferences are important sources of measurement errors, we expect them to be less significant when analyzing *S*_*i*_^*L*^. All the other indices are based on the follow-up question, in which respondents can embellish their factual living situation by stating that items are missing for other reasons. If factual living standards improve significantly in certain life domains (say, housing), average deprivation may nevertheless remain quite stable when using *S*_*i*_^*P*^, because the missing items of the fewer people still lacking these more prevalent items are weighted higher. All in all, however, we assume the results to be quite similar across different indices, because research has show that they are all highly correlated (Bosch 2001; Lipsmeier 1999).^13^

### Regression models

Using sum indices, our dependent variable “deprivation” is a count. The Poisson distribution is a suitable model for counts (even when they include non-integer values such as *S*_*i*_^*N*^ and *S*_*i*_^*P*^), but is a rather inflexible distribution because its dispersion is fixed to its mean. An extension, the negative binomial (NB) distribution, allows overdispersed data (Cameron and Trivedi 2013). Usually, differences between the parameter estimates of both types of regression models are small. We use a log-linear regression model that relates the conditional expected mean count, *μ*_*i*_, to the independent variables and estimate its parameters with maximum likelihood assuming that the counts are distributed according to the NB distribution:

3 $$\log \left( {\mu_{i} } \right)\, = \,\beta_{0} \, + \,\mathop \sum \limits_{k} \gamma_{k} X_{ki} \, + \,\beta_{1} t\, + \,\beta_{2} R_{i} \, + \,\beta_{3} R_{i} \left( {t\, - \,5} \right)\, + \,\beta_{4} M_{i} \, + \,u_{i}$$

*u*_*i*_ is a random effect for each individual *i* that is assumed to be Gamma distributed (exp(*u*_*i*_) ∼ γ(1/*α*, *α*)). The larger the Gamma parameter *α*, the larger the overdispersion (when *α* = 0 the NB reduces to the Poisson distribution).^14^

In order to test all our hypotheses in one model, our analysis uses the original samples 1 and 2 (being observed 2006–2013) and pools them with the refreshment samples 6 and 7 (being observed 2011–2013). We estimate (3) seperately for the resident population (samples 2 and 6) and for the stock of UBII recipients at the time of the interview from samples 1 and 7 (both called “subgroups” in the following).

All estimations are done in three different versions:

- Version A1 uses the cross-sectional weights provided with the data and specifies a clustered and stratified survey design (Trappmann 2013a, b). These (calibrated) weights control different selection probabilities due to the survey design and different patterns of non-response (among them panel attrition).^15^ Panel attrition can also be controlled by using a balanced panel (Version A2). We use this option for the subgroup from the resident population.^16^
- Version B proceeds in a similar way, except that it uses only the design weights capturing different selection probabilities due to the survey design, but ignoring different patterns of non-response (and panel attrition).
  If panel attrition (H6) is one cause of a decreasing deprivation trend, it should be smaller with the Version A estimates.
- Version C estimates model (3) with respondent-specific fixed effects (FE) and hence, controls for all observed *and unobserved* time-constant characteristics of the respondents.^17^ In doing so, it allows to control for selection and confounding biases due to unobserved time-constant characteristics. Version C estimates do not control the survey design and also do not use weights. Moreover, to ease computation, FE Poisson models are used instead of FE NB regression models.

According to H1, material deprivation is assumed to be the result of insufficient resources and behaviors. Therefore, we only want to compare individuals having the same behaviors and resources. In model (3) they are represented by the *X*_*k*_. They are explained in greater detail in the following section. The main parameter of interest in model (3) is the linear time trend *β*_1_ ($t = 1, \ldots , 7$. identifies the single panel waves).^18^ It is expected to be zero after controlling for resources and behaviors, i.e., after testing H1.

If it is not, Sect. 3 has mentioned several measurement and selectivity problems that may generate a seemingly negative time trend. To control for them, model (3) includes a dummy variable *R*_*i*_ indicating whether the respondent is member of the refreshment samples 6 and 7 (*R*_*i*_ = 1) or the original samples 1 and 2 (*R*_*i*_ = 0). If H2a is true (respondents economizing on interview time), the parameter *β*_2_ should be positive in both subgroups, but according to H2b the difference between original and refreshment samples should gradually diminish and therefore, the interaction parameter *β*_3_ should be again negative in both subgroups. Moreover, we include a dummy variable *M*_*i*_ indicating survey mode with *M*_*i*_ = 1 (CAPI) and *M*_*i*_ = 0 (CATI). If H3 is true (interviewers economizing on interview time), the parameter *β*_4_ should be negative. If H4 is true and selection of specific individuals into CAPI mode plays a role, the parameter *β*_4_ should be positive. The estimate $\hat{\beta }_{4}$ will show the net effect of H3 and H4. Finally, if more deprived individuals leave the panel and H6 is true, weighted analyses controlling for panel attrition should provide a less negative time trend *β*_1_ than unweighted analyses. The same should be true if we exclude panel attrition and focus only on respondents that participated in all panel waves.

We are stumped if after all these tests the linear time trend *β*_1_ is still negative and significant. Formally, without any controls, *β*_1_ is a mixture of age, cohort, and period effects. The controls *X*_*k*_ include dummy variables indicating the birth cohort of the respondents. Nevertheless, with birth cohort as a control, *β*_1_ still is a mixture of age and period effects. Adaptive preferences are examples of such ageing effects. But we assumed them to occur predominantly in the subgroup of UBII recipients (according to H5 *β*_1_ may be negative for UBII recipients). Unfortunately, as our following estimations will show, *β*_1_ remains negative and significant also in the subgroup from the resident population. This calls for possible explanations in terms of period effects. Changes in the broader economic and societal context are examples of such effects. We defer the discussion of possible context explanations for a decreasing deprivation trend to the concluding section.

### Controls and number of cases

We only want to compare individuals having the same behaviors and resources in our analysis and therefore, have to control for them. The variables *X*_*k*_ include log equivalized household income in 2006 prices (continuous variable),^19^ household type (5 dummies), language used during the interview (1), residence in West or East Germany (1), UBII receipt at the interview date (1), number of UBII receipts at previous interview dates (count), age group of the reference person reporting on deprivation (5 dummies), gender (1), birth cohort (4), school leaving certificate (7), and training qualification of the respondent (10) (for more details see Tables S2, S3 in Additional file 1). Unfortunately, data on (housekeeping) behaviors are not available in PASS. As long as these behaviors (as well as other unmeasured determinants of deprivation) remain constant within the observation period, we can control for them using respondent-specific fixed effects in the regression model. Altogether, 12,884 respondents from the resident population samples (contributing 42,448 observations in the observation period) and 9220 respondents from the UBII recipient samples (contributing 21,242 observations) had valid values on all variables and were used to estimate model (3) parameters in both subgroups.^20^ Respondents visiting a school or receiving vocational training were excluded from the analysis.

## Results

**Fig. 2:**
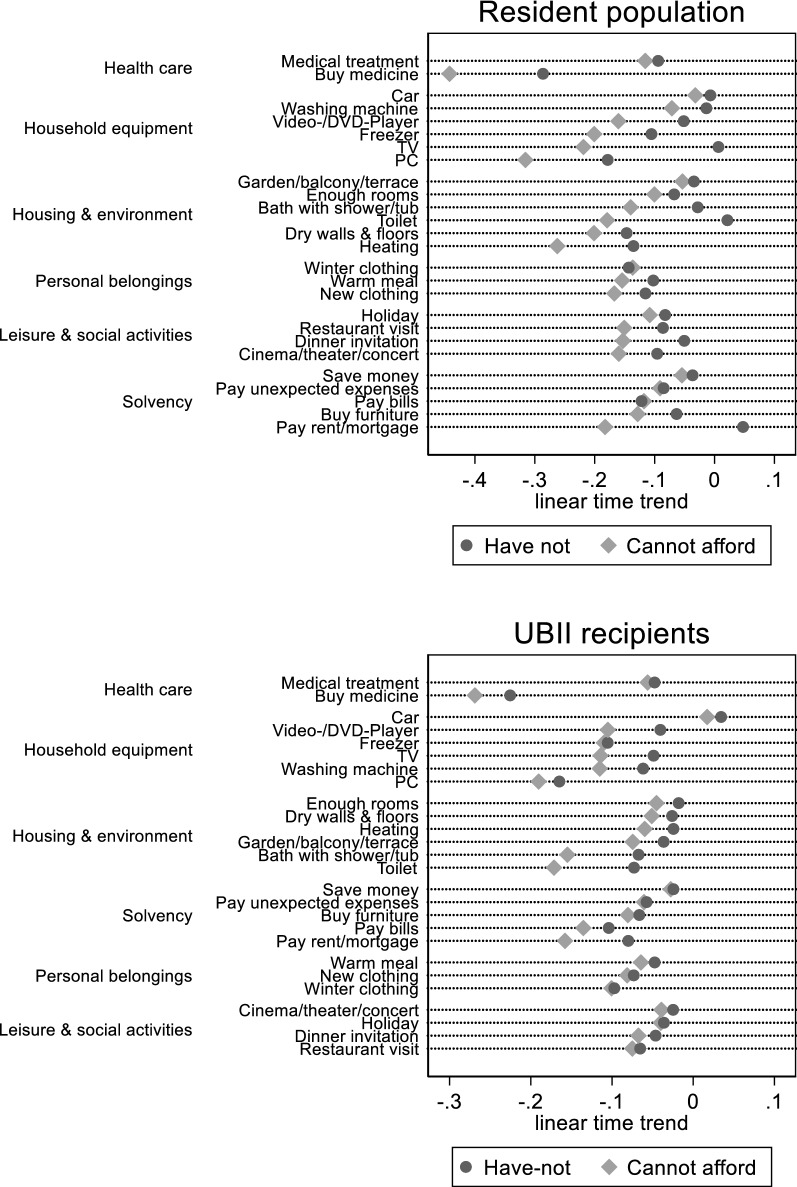
Linear time trend of have-nots and unaffordable items (logistic regression coefficients). Data: Panel study labor market and social security (PASS) samples 1, 2, 6, and 7; waves 1–7, 2006–2013. Logistic regression models controlling for survey design, selection probabilities, and non-response

As already mentioned, average real incomes and employment increased during our observation period. The improved economic conditions are also mirrored in our sample of the resident population. In Sample 2, average real equivalized household incomes (measured in 2006 prices) raised from 1412 € in 2006 to 1810 € 2013—a 28.2% increase. Relative income poverty decreased slightly from 15.7 to 14.0% and absolute income poverty (using the 2006 poverty threshold in all years) dropped significantly from 15.7 to 12.4%.^21^ Correspondingly, the prevalence of all the items of the PASS deprivation instrument increases in the observation period. Using simple logistic regression models (not using the controls mentioned in Sect. 4.3), we estimated a linear time trend for the two dichotomous variables *F*_*ij*_ (“cannot afford”) and *L*_*ij*_ (“not have”) for each item. Figure 2 shows the estimated trend parameters, which—with some exceptions—are all negative. Some particular items show remarkable negative trends (e.g., the PC or being able to buy medicine that is not paid by the health insurance), but overall no specific life domain stands out. Also the items that one would expect to decrease due to technological change and housing improvements (except the PC) show trends not much different from other items.

Certainly, the economic situation of respondents that receive UBII at the time of the interview (Sample 1) is not that positive. Relative poverty increases from 67.8 to 75.4% in the observation period and absolute poverty stays more or less the same, with around two-thirds of the respondents being poor according to the poverty threshold in 2006. But we also see improvements: Average real equivalized household incomes, although on a much lower level, rise from 642 € in 2006 to 808 € in 2013, which is also a significant 25.9% increase. Of course, it is an average increase, but improvements can be seen in all parts of the UBII recipients’ income distribution, especially so at the lower end (the first quartile increases by 42%, the second by 28%, and the third by 23%). Nevertheless, given the much lower income level and much higher poverty rate, it is surprising that we observe decreasing missingness for all items, very similar to the sample of the resident population (see Fig. 2). Again no particular life domain stands out, for which the have-nots and unaffordable items decrease relatively much.

Model (3) allows a more precise test of a decreasing deprivation trend than the unconditional trend estimates in Fig. 2, because it controls for the socio-economic profiles of the respondents and potential measurement and selection problems as discussed in Sect. 3. Table 1 shows only the estimates of the main parameters $\hat{\beta }_{1}$  −  $\hat{\beta }_{4}$.^22^ Independent of the specific deprivation index, subgroup, or estimation method, the linear trend $\hat{\beta }_{1}$ is significant and negative. It may be argued that our list of controls is incomplete. For example, it has been discussed that certain behaviors, health statuses or over-indebtedness are possible explanations for the weak association between income and deprivation (Perry 2002). However, the estimated negative time trend does not disappear when using FE estimates (Version C estimates), i.e., when controlling for all time-constant observed *and unobserved* characteristics of the respondents. Hence, these characteristics that have not been controlled in our analysis cannot be used as an argument against the estimated negative time trend, as long as these characteristics remain constant over time. In other words, model (3) provides no support for H1; especially so, because model (3) takes care of all the alternative explanations for a negative time trend we have discussed in Sect. 3. In the following we will have a brief look at these measurement and selection problems.

**Table 1:** Determinants of material deprivation (four deprivation indices, log-linear regression coefficients)

Independent variables	Cannot afford				Cannot afford (weighted with necessities)				Cannot afford (weighted with prevalences)				Have not			
	A1	A2	B	C	A1	A2	B	C	A1	A2	B	C	A1	A2	B	C
*Resident population*																
*t*	− 0.042***	− 0.052***	− 0.046***	− 0.109***	− 0.051***	− 0.060***	− 0.063***	− 0.104***	− 0.019*	− 0.024***	− 0.021*	− 0.081***	− 0.026***	− 0.025***	− 0.028***	− 0.037***
	(0.010)	(0.007)	(0.010)	(0.003)	(0.011)	(0.008)	(0.010)	(0.007)	(0.010)	(0.007)	(0.010)	(0.004)	(0.004)	(0.003)	(0.004)	(0.002)
Sample wave 1	Reference	Reference	Reference	Reference	Reference	Reference	Reference	Reference	Reference	Reference	Reference	Reference	Reference	Reference	Reference	Reference
Refreshment sample wave 5	− 0.030	0.019	− 0.054	0	0.034	0.111*	0.053	0	0.007	0.063	− 0.007	0	− 0.011	− 0.012	0.006	0
	(0.080)	(0.046)	(0.070)		(0.088)	(0.052)	(0.071)		(0.081)	(0.044)	(0.068)		(0.038)	(0.017)	(0.030)	
*t* * Refreshment sample wave 5	0.022	− 0.030	0.005	− 0.002	− 0.062	− 0.077*	− 0.060	− 0.037	− 0.028	− 0.069*	− 0.039	− 0.037*	0.012	0.017	0.007	0.021*
	(0.053)	(0.033)	(0.038)	(0.017)	(0.061)	(0.038)	(0.044)	(0.035)	(0.055)	(0.032)	(0.038)	(0.021)	(0.023)	(0.012)	(0.020)	(0.010)
CATI	Reference	Reference	Reference	Reference	Reference	Reference	Reference	Reference	Reference	Reference	Reference	Reference	Reference	Reference	Reference	Reference
CAPI	0.010	− 0.033	− 0.038	− 0.013	0.155***	0.079**	0.106*	− 0.004	0.054	− 0.009	Reference	− 0.013	0.063***	0.042***	0.045**	0.003
	(0.050)	(0.027)	(0.057)	(0.024)	(0.049)	(0.030)	(0.047)	(0.049)	(0.049)	(0.026)	(0.054)	(0.031)	(0.018)	(0.010)	(0.018)	(0.017)
Constant	11.385***	12.228***	11.410***		5.553***	5.800***	5.754***		9.869***	10.744***	9.911***		5.146***	5.247***	5.310***	
	(0.398)	(0.252)	(0.385)		(0.342)	(0.180)	(0.333)		(0.380)	(0.234)	(0.353)		(0.141)	(0.080)	(0.146)	
ln(Dispersion parameter)	0.216***	0.392***	0.354***	0	− 2.547***	0	− 2.298***	0	− 0.300***	− 0.190***	− 0.184***	0	− 2.544***	− 2.503***	− 2.270***	0
	(0.040)	(0.022)	(0.038)		(0.266)		(0.224)		(0.049)	(0.030)	(0.047)		(0.090)	(0.048)	(0.084)	
Observations	42,448	19,650	42,101	26,055	42,448	19,650	42,101	26,055	42,448	19,650	42,101	26,055	42,448	19,650	42,101	38,030
Individuals	12,884	3741	12,706	5930	12,884	3741	12,706	5930	12,884	3741	12,706	5930	12,884	3741	12,706	8948
*UBII recipients*																
*t*	− 0.063***		− 0.059***	− 0.058***	− 0.062***		− 0.059***	− 0.058***	− 0.043***		− 0.038***	− 0.043***	− 0.034***		− 0.036***	− 0.026***
	(0.009)		(0.008)	(0.009)	(0.010)		(0.009)	(0.016)	(0.010)		(0.008)	(0.011)	(0.005)		(0.005)	(0.008)
Sample wave 1	Reference		Reference	Reference	Reference		Reference	Reference	Reference		Reference	Reference	Reference		Reference	Reference
Refreshment sample wave 5	0.095*		0.081*	0	0.074		0.062	0	0.118**		0.102**	0	0.039		0.049*	0
	(0.041)		(0.036)		(0.048)		(0.044)		(0.043)		(0.038)		(0.026)		(0.024)	
*t* * Refreshment sample wave 5	− 0.034*		− 0.010	0.003	− 0.056*		− 0.023	− 0.006	− 0.067***		− 0.041*	− 0.030*	− 0.019		− 0.006	0.003
	(0.019)		(0.017)	(0.012)	(0.024)		(0.021)	(0.023)	(0.021)		(0.018)	(0.015)	(0.013)		(0.012)	(0.011)
CATI	Reference		Reference	Reference	Reference		Reference	Reference	Reference		Reference	Reference	Reference		Reference	Reference
CAPI	0.116***		0.077***	0.073***	0.134***		0.091***	0.095***	0.122***		0.081***	0.078***	0.073***		0.050***	0.030*
	(0.017)		(0.016)	(0.015)	(0.020)		(0.019)	(0.028)	(0.018)		(0.017)	(0.019)	(0.011)		(0.010)	(0.014)
Constant	3.194***		3.221***		2.014***		1.996***		2.758***		2.756***		3.117***		3.145***	
	(0.123)		(0.112)		(0.134)		(0.123)		(0.124)		(0.115)		(0.068)		(0.076)	
ln(Dispersion parameter)	− 2.141***		− 2.221***		− 46.538		− 24.021		− 2.766***		− 2.920***		0		0	
	(0.067)		(0.068)						(0.108)		(0.122)					
Observations	21,242		21,108	16,822	21,242		21,108	16,822	21,242		21,108	16,822	21,242		21,108	16,863
Individuals	9220		9142	4823	9220		9142	4823	9220		9142	4823	9220		9142	4841

Data: Panel Study Labor Market and Social Security (PASS) samples 1, 2, 6, and 7; waves 1–7, 2006–2013

Only respondents from households receiving UBII payments at the time of the interview. A1: Negative binomial regression models controlling for survey design, selection probabilities, and non-response. A2: Negative binomial regression models controlling for panel attrition with a balanced panel. B: Negative binomial regression models controlling for survey design and selection probabilities. C: FE Poisson regression models. * p<.05, ** p<.01, *** p<.001 (one-sided tests)

The estimated main effect $\hat{\beta }_{2}$ of the refreshment sample is positive in the subgroup of UBII recipients, but not significant for every estimation method and not for the index *S*_*i*_^*N*^.^23^ This shows that respondents answering the PASS deprivation instrument for the first time report much more deprivation than respondents having answered (“learned to deal with”) the instrument in previous panel waves. In principle, this corroborates H2a for UBII recipients. Moreover, in accordance with our expectations, this effect occurs independent of whether we focus on unaffordable items only or on have-nots including items missing also for other than financial reasons. Finally, according to H2b, the effect diminishes over time: The estimate of the interaction effect $\hat{\beta }_{3}$ is negative (although not always significant, possibly because the refreshment sample was observed only three waves).

The overall conclusion is that over time UBII recipients report differently about their material deprivation (i.e., mentioning less unaffordable items and fewer have-nots) and we cannot rule out the possibility that this occurs even when their factual situation does not improve. However, it is surprising that one observes this effect only among UBII recipients. The estimate of the main effect $\hat{\beta }_{2}$ and the estimate of the interaction $\hat{\beta }_{3}$ are hardly significant in the subgroup from the resident population. This is no strong support for H2 in the subgroup from the resident population. The fact that it occurs only in the subgroup of UBII recipients supports H5, which assumes that predominantly UBII recipients adapt their preferences.

We also assumed survey mode to be associated with the level of deprivation, however, with two conflicting hypotheses concerning CAPI mode: a negative interviewer effect (H3) and a positive selection effect (H4). It seems as if the positive selection effect prevails in the subgroup of UBII recipients, because all estimates $\hat{\beta }_{4}$ of the survey mode effect (CAPI) are positive and significant. If one weights unaffordable items by their necessities (index *S*_*i*_^*N*^) or only counts the have-nots (index *S*_*i*_^*L*^), the mode effect is also significantly positive in the subgroup from the resident population, but when using FE estimation (Version C) $\hat{\beta }_{4}$ loses its significance. In sum, more deprived UBII recipients are disproportionally interviewed in CAPI mode, which supports H4 for this subgroup. For the subgroup from the resident population the conclusion is less clear.

Finally, we assumed that panel attrition might explain decreasing deprivation when the worst-off individuals leave the panel (H6). As can be seen from Table 1, the estimates $\hat{\beta }_{1}$ remain significant if weights that control for non-response (including attrition) (Version A (A1) estimates) or a balanced panel (Version A2 estimates) are used. Therefore, to the extent that the weights (and balancing) control for the assumed selection process, the possibility can be ruled out that panel attrition drives decreasing deprivation.

## Discussion

We asked whether material deprivation is decreasing in Germany. Seven waves from the German Panel Study Labor Market and Social Security (PASS), covering the period from 2006 to 2013, were used to answer this research question. During the observation period, Germany experienced a significant increase in average real incomes and employment. However, a more differentiated analysis demonstrated that the returns of this economic upswing were mostly reaped in the upper segments of the income distribution, while the middle stagnated and lower segments lost (Grabka and Goebel 2017). Nevertheless, looking at the PASS sample of the resident population, we observed a slight decrease in relative income poverty and even more importantly, a significant decrease in absolute income poverty (as measured by the fixed AROP).

We described and discussed the PASS instrument to measure material deprivation. It is the only large scale German survey providing such a comprehensive measure (plus it also measures perceived necessities of the deprivation items in the population). Although in a way a unique measurement instrument, it is also not without problems. Generally speaking, measuring material deprivation is almost as difficult as measuring income and prone to measurement errors and selection biases. In our analysis we controlled for possible incentives for respondents and interviewers to economize on a complicated and time-consuming instrument such as the one on deprivation. We assumed that CAPI gives interviewers more possibilities to economize than CATI and that respondents, after having answered the instrument in previous waves and seen how many answers it entails, may be inclined to not report have-nots in the following waves, because that would result in lengthy follow-up questions. In the sample of the resident population we found no strong signs of such measurement errors. We also could not find any signs of selection bias due to panel attrition. But we found clear evidence for decreasing material deprivation in the observation period, not only in overall indices of deprivation, but also in sub-indices focusing on specific life domains. Since this trend corresponds to the decreasing trend in absolute income poverty, we agree with Groh-Samberg and Goebel (2007) who conclude that deprivation measures are similar to absolute poverty measures if the list of possessions and activities is not updated to changed living standards in society.^24^

In other words: Deprivation measures do not only tell us cross-sectionally how different the materially deprived population is from the people having low incomes, they also measure longitudinally which people dispose of a given minimum set of commodities and which people are deprived of this minimum. In principle, the concept of deprivation is a relative one: “Deprivation may be defined as a state of observable and demonstrable disadvantage relative to the local community or the wider society or nation” (Townsend 1987, p. 125). However, if it is measured with a time-constant list of commodities and if this measurement is time-invariant, its measurement will behave like an absolute standard.

Our analysis provided also some unexpected results that need further investigation. Next to an analysis of the resident population, we looked at people in employment age with basic income support (UBII recipients) who, by definition, command only few economic resources. UBII payments are based on the so-called “Regelbedarf”, which is the necessary livelihood defined as ensuring the socio-cultural subsistence level in Germany. For people living at this officially defined subsistence level one would not expect decreases of material deprivation over time. But our analysis of a sample of UBII recipients found a decrease similar to the decrease in the resident population, although on a higher level (UBII recipients are surely more deprived than the average resident in Germany). There are indications that—at least on average—economic conditions improved also in this group in the observation period:

1. Housing costs are compensated separately and improvements in housing conditions are taken over by the authorities as long as size and quality of the accommodation do not exceed the standards for UBII recipients. Hence, less deprivation with respect to the items concerning housing and living environment is possible.
2. Single parents are awarded additional payments for certain needs which may even lift them out of income poverty.
3. The group of individuals receiving work incomes besides UBII payments has increased steadily over time and they are a group of UBII recipients possessing relatively large incomes.

With respect to income, we have seen improvements in all parts of the UBII recipients’ income distribution (although on a very low level). But are these improvements large enough to explain such a significant decrease in material deprivation? Absolute income poverty, as measured by the fixed AROP, stayed more or less the same in this group!

This question is difficult to answer, because our analysis of the UBII recipients also provided evidence that measurement equivalence over time is at stake in this sample. Compared to UBII recipients who had answered the deprivation instrument in previous waves, new entrants into the UBII sample reported not only more have-nots, but also more financial reasons for their absence. In other words, at first contact UBII recipients seem to report more deprivation. Since we cannot rule out the possibility that this may be an overstatement, or the less frequent reporting at following contacts an understatement, it is unclear how to interpret the overall decrease in material deprivation among UBII recipients.

Since the effect occurs only among UBII recipients, it may be an effect of adaptive preferences which we assumed to play a role for this subgroup (see H5). If preferences change over time, a subjective poverty measure such as the respondents’ *own* assessment of their living standard (compared to an objective assessment by an external observer) will underestimate the incidence of deprivation (Halleröd 2006). Changing preferences can only be excluded, if every year *new* members of the UBII population are interviewed and not the same UBII recipients repeatedly over time. In fact, if one pools the first contact interviews from all UBII samples in PASS (i.e., the original, refreshment, and replenishment samples) and again estimates model (3), the linear time trend gets considerably smaller and often loses its significance (see Table S5 in Additional file 1). The remaining small, but significant $\hat{\beta }_{1}$ possibly reflect the slight economic improvements we have mentioned for UBII recipients before. This observation definitely needs more investigation. In any case, adaptive preferences are a severe challenge for the repeated application of item lists in deprivation research.

## Additional file

**Additional file 1.** Additional tables.

## Footnotes

1 The population for Sample 7 is defined as all households with at least one benefit unit in July 2010.

2 In case of winter clothing it was asked whether *each* household member has sufficient winter clothing. For other items this specification was missing. However, it can be assumed that a washing machine, a computer, or a car (and all the other items) are shared by all household members.

3 For some items the questionnaire asked whether *all* household members were doing the respective activity (e.g., a holiday trip). In these cases, interviewers were advised to code a negative answer, if only some but not all household members were doing the activity.

4 Note that PASS uses a stratified cluster design with households at the very end of the multi-stage sampling procedure. Such a complex survey design cannot be easily handled with robust standard errors.

5 However, wave 11 of PASS (to be available in autumn 2018) contains a new module on financial literacy and behavior.

6 Empirical data on these additional selection mechanisms is not available. With respect to income, our analysis sample (see below) shows significantly lower real equivalized household incomes for German residents interviewed in CAPI mode. They are on average 190.75 € lower than in CATI mode. For UBII recipients the differences between CAPI and CATI is not significant. Real equivalized household incomes from CAPI interviews are on average only 24.87 € lower.

7 In our analysis sample (see below) max. 66.8% of the German residents and max. 53.5% of the UBII recipients were interviewed in CAPI mode in all the years they participated in the panel.

8 Samples 1 and 7 already provide a large enough sample size to test our hypotheses for the subgroup of UBII recipients. Including the UBII refreshment samples would necessitate a more complicated model to test H2b.

9 The generated variable *alg2abez* is used for this selection. It is provided with the data and identifies households in which at least one person receives UBII benefits.

10 Both dummy variables have missing values when item *j* has not been answered. *F*_*ij*_ is also missing when other reasons are mentioned, while in case of *L*_*ij*_ the category *L*_*ij*_ = 1 includes individuals that cannot afford the item, individuals lacking the item for other reasons, *and* individuals not answering the follow-up question.

11 There are 10 out of the 26 items having a discrimination parameter *a*_*j*_. of at least 2.7. They include all items from the domains “leisure and social activities”, “personal belongings”, and “health care” plus 2 out of 5 items from the domain “solvency”. On the other side, all items from the domains “household equipment and devices” and “housing and living environment” do not belong to this group.

12 *p*_*j*_ was computed counting the zeroes of the dichotomous variable *L*_*ij*_. as a proportion of all valid values for each item in each wave. Therefore, *p*_*j*_ is changing over time. In wave 1, each member of Sample 1 had to rate the necessity of six randomly selected items from the PASS instrument. The random selection assured that each item was rated (although by a random sample from Sample 1). *n*_*j*_ equals the proportion of respondents rating item *j* on a 3-point scale as “absolutely necessary”. These proportions were carried over to the following waves, assuming no change in perceived necessities in the observation period. Hence, *n*_*j*_. is a time-constant variable.

13 For the Sample 1 (stock of UBII recipients) the indices *S*_*i*_^*F*^, *S*_*i*_^*N*^, and *S*_*i*_^*P*^. correlate with *r* > 0.94. and *S*_*i*_^*L*^ correlates with *S*_*i*_^*F*^, *S*_*i*_^*N*^, and *S*_*i*_^*P*^ with *r* > 0.85. The corresponding correlation coefficients for Sample 2 (resident population) are *r* > 0.94 and *r* > 0.72.

14 All estimations are done with Stata 15.1. Some fail to converge due to an almost zero dispersion parameter and are replaced by Poisson regression models.

15 These weights are also used for all descriptive analyses (average incomes, poverty rates, figures).

16 It is not useful for our definition of the subgroup of UBII recipients. If we only include respondents receiving UBII benefits at the time of the interview, a balanced panel would result in a highly selective group of continuous (long-term) UBII recipients.

17 In case of UBII-recipients, the FE analyses—like all the other analyses of UBII recipients—include only respondents that receive UBII payments at the time of the interview.

18 In developing this model we also specified a non-parametric time trend using wave dummies. Restricting the estimated dummy effects to be a linear function of *t* never provided a worse fit of the model, which legitimates our choice of a linear trend function.

19 The data on monthly household incomes are provided with the data. They also include equivalized household incomes. We use the variable *oecdincn* that applies the new OECD equivalence scale and deflate the equivalized household incomes with the consumer price index from the German Statistical Office. Unfortunately, the income data include some extremely large income values, also in the samples of UBII recipients. In the absence of any information on the plausibility of these values we decided to use the log-transformation in order to downsize the large values and make the distribution more symmetric.

20 Version B and C estimates are based on smaller sample sizes because design weights are missing for individuals entering later into the panel (B) or for individuals not changing their characteristics over time (C). Naturally, using a balanced panel (A2) also reduces the sample size.

21 The wave-specific poverty thresholds are based on real equivalized household incomes from all (original, refreshment, and replenishment) samples in PASS during the observation period. The threshold in wave *t* equals 60% of median real equivalized household incomes in wave *t*. Weighted data (Version A1) are used for this computation.

22 Tables S2, S3 in Additional file 1 show the complete list of estimates including the estimated effects $\hat{\gamma }$ of the controls, all of which are very much in line with what one would expect for each control variable. In various robustness tests (not shown) we have also tested whether the estimated effects $\hat{\beta }$ remain stable in both subgroups when using domain-specific deprivation indices or when selecting only the “best” items according to the item response model (1). These robustness tests showed no substantively different results from the ones using the complete list of items.

23 Note that version C estimates use only the within-variation and therefore, the effects of time-constant independent variables, such as being member of the refreshment sample, are not identified.

24 In fact, if we include this subgroup’s fixed AROP as a context characteristic into model (3), it has the largest positive effect among all effect estimates and the linear time trend gets considerably smaller and often loses its significance (see Table S4 in Additional file 1).
